# Cellular Development Associated with Induced Mycotoxin Synthesis in the Filamentous Fungus *Fusarium graminearum*

**DOI:** 10.1371/journal.pone.0063077

**Published:** 2013-05-07

**Authors:** Jon Menke, Jakob Weber, Karen Broz, H. Corby Kistler

**Affiliations:** 1 Department of Plant Pathology, University of Minnesota, St. Paul, Minnesota, United States of America; 2 Molekulare Phytopathologie, Universität Hamburg, Hamburg, Germany; 3 USDA ARS Cereal Disease Laboratory, St. Paul, Minnesota, United States of America; Soonchunhyang University, Republic of Korea

## Abstract

Several species of the filamentous fungus *Fusarium* colonize plants and produce toxic small molecules that contaminate agricultural products, rendering them unsuitable for consumption. Among the most destructive of these species is *F. graminearum,* which causes disease in wheat and barley and often infests the grain with harmful trichothecene mycotoxins. Synthesis of these secondary metabolites is induced during plant infection or in culture in response to chemical signals. Our results show that trichothecene biosynthesis involves a complex developmental process that includes dynamic changes in cell morphology and the biogenesis of novel subcellular structures. Two cytochrome P-450 oxygenases (Tri4p and Tri1p) involved in early and late steps in trichothecene biosynthesis were tagged with fluorescent proteins and shown to co-localize to vesicles we provisionally call “toxisomes.” Toxisomes, the inferred site of trichothecene biosynthesis, dynamically interact with motile vesicles containing a predicted major facilitator superfamily protein (Tri12p) previously implicated in trichothecene export and tolerance. The immediate isoprenoid precursor of trichothecenes is the primary metabolite farnesyl pyrophosphate. Changes occur in the cellular localization of the isoprenoid biosynthetic enzyme HMG CoA reductase when cultures non-induced for trichothecene biosynthesis are transferred to trichothecene biosynthesis inducing medium. Initially localized in the cellular endomembrane system, HMG CoA reductase, upon induction of trichothecene biosynthesis, increasingly is targeted to toxisomes. Metabolic pathways of primary and secondary metabolism thus may be coordinated and co-localized under conditions when trichothecene biosynthesis occurs.

## Introduction

Filamentous fungi are capable of producing a wide spectrum of ancillary small molecules. The functional significance of small molecule synthesis by fungi is generally unknown but often these secondary metabolites have been associated with novel biological properties such as antibiotic, antitumor, toxic or hormonal activities [1], [2]. Secondary metabolites are structurally diverse but tend to be based on polyketide, terpenoid or non-ribosomal peptide structural components [3]. Fungal genome sequencing projects have uncovered a surprisingly large number of genes for polyketide synthases, terpenoid synthases and non-ribosomal peptide synthases potentially encoding a previously unrecognized abundance of novel secondary metabolites (*e.g.* [4], [5]). While the genetics and biosynthetic enzymology of the best-known fungal secondary metabolites are well documented, relatively little is known about the subcellular localization and cellular machinery required for assembly of these molecules.

The filamentous fungi *Acremonium chrysogenum* and *Aspergillus nidulans* each synthesize amino acid derived ß-lactam metabolites (penicillin and cephalosporin, respectively) and the biosynthetic enzymes, pathway precursors and intermediates, and transporters for the ß-lactams have been placed variously in Golgi-derived vesicles, vacuoles, peroxisomes and the cytoplasm [6], [7], [8], [9], [10], [11]. In *Aspergillus*, biosynthetic enzymes implicated in the production of the polyketide-derived furanocoumarin aflatoxin have been localized to peroxisomes, vesicles and vacuoles. Peroxisomes have been reported to supply part of the acetyl-CoA used for aflatoxin biosynthesis [12]. Nor-1, implicated in an early step of aflatoxin biosynthesis in *A. parasiticus*, is present in the cytoplasm of toxigenic cells, though localization of Nor-1 to the vacuole coincides with high rates of aflatoxin biosynthesis [13]. Similar localization patterns were observed in the study of aflatoxin biosynthetic enzymes OmtA and Ver-1 [14], [15]. Chanda and associates [16] suggest the aflatoxin metabolic pathway flows from peroxisomes to aflatoxisomes that export the toxin from the cell.

Trichothecenes are isoprenoid metabolites produced by species of *Fusarium*, *Myrothecium*, *Stachybotrys* and other ascomycetous fungi in the order Hypocreales. Toxic trichothecenes such as deoxynivalenol (DON) may contaminate grain infected with any of several *Fusarium* species. The U.S. Food and Drug Administration and international regulatory agencies have placed limits on DON levels in grain and products made from grain [17]. While much of the genetics and enzymology of trichothecene biosynthesis in *Fusarium* have been described within the past two decades [18], the cellular processes involved in its synthesis and export remain to be elucidated. In this study, we sought to define the cellular localization of two enzymes in the trichothecene biosynthetic pathway and a transporter involved in DON tolerance in *F. graminearum*. Our observations led us to develop hypotheses on the coordination of primary and secondary metabolism in toxigenic cells and to suggest new ideas for facilitated export of trichothecenes from cells.

## Results

### Cellular Co-localization of Two Enzymes of the Trichothecene Biosynthetic Pathway

To determine where proteins responsible for trichothecene biosynthesis are localized within the cell, two enzymes were tagged at their C-termini with a fluorescent protein. Both FgTri4p and FgTri1p are cytochrome P450 oxygenases [19], each with a single membrane anchoring domain. Tri4p is responsible for converting trichodiene to the tri-oxygenated intermediate isotrichodermol [20], whereas Tri1p is responsible for both C-7 and C-8 hydroxylation of the trichothecene molecule in *F. graminearum* [21]. As such, FgTri4p and FgTri1p, respectively, catalyze the second and sixth step of the predicted trichothecene reaction pathway that consists of seven enzymatic reactions [22]. FgTri1p and FgTri4p were singly or doubly tagged with their carboxy termini fused to GFP or RFP in the wild type *F. graminearum* strain PH-1.

**Figure 1:**
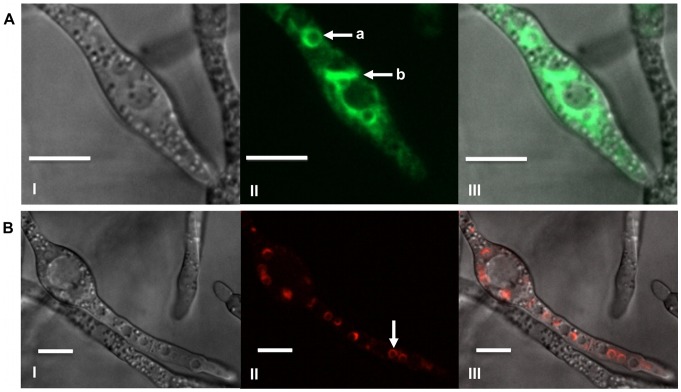
Localization of Tri1p and Tri4p in toxin induced cells. A) Tri1p::GFP localized to a stationary spherical organelle (a) and a membranous network (b) within the cytoplasm of strain PH-1Tri1::GFPA. Confocal bright field DIC (I); GFP (II); and GFP and DIC (III) overlay images of cells are shown. B) Localization of Tri4p::RFP to a stationary spherical organelle (arrow) within the cytoplasm of strain Tri4::RFPB. Confocal bright field DIC (I); RFP (II); and RFP and DIC overlay images are shown. Both strains were incubated in TBI medium for 36 h. Scale bar = 10 µm.

As previously noted, during induction of trichothecene biosynthesis in liquid TBI medium [23], morphological differentiation occurs as characterized by subapical hyphal swelling, formation of ovoid toxigenic cells and extensive hyphal branching and thickening. Fluorescence of GFP in Tri1::GFP strains was initially observed in ovoid cells within the mycelium (Figure 1A); though GFP fluorescence progressively became evident in cells adjacent to these structures and among contiguous cells as cultures aged. Tri1p::GFP fluorescence localized to the periphery of stationary spherical structures approximately 3 to 4 µm in size and was also evident in a membranous network within the cytoplasm of cells.

Localization of RFP fluorescence in Tri4::RFP strains also was initially observed in swollen ovoid cells within mycelia induced for trichothecene biosynthesis and progressively in adjacent cells within ageing cultures (Figure 1B). Because Tri4p::RFP fluorescence also localized to the periphery of stationary spherical structures of similar size, doubly tagged PH-1Tri1::GFP/Tri4::RFP strains were created to test whether Tri1 and Tri4 proteins were targeted to the same organelle.

**Figure 2:**
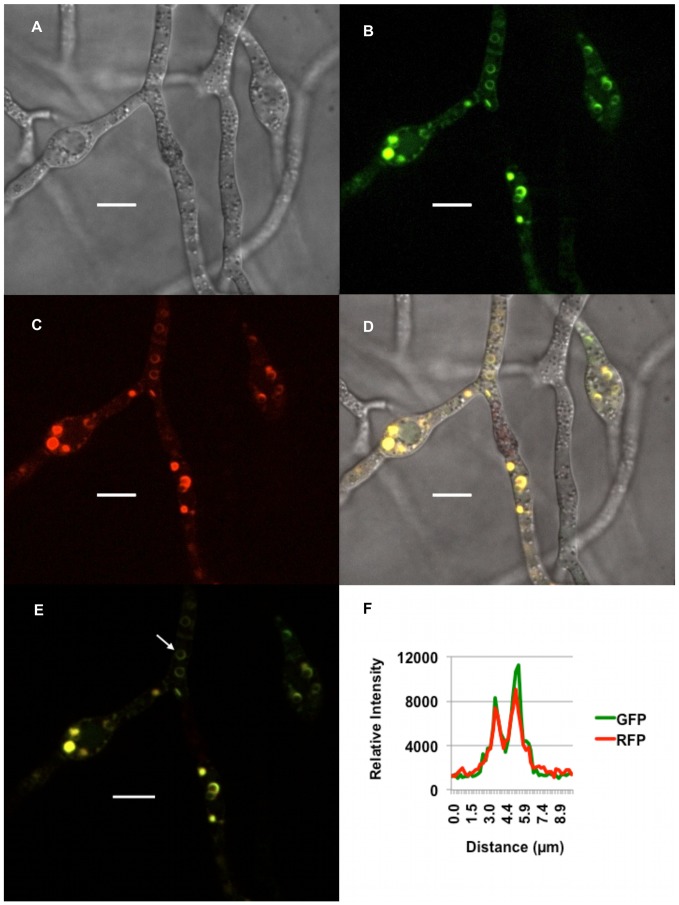
Co-localization of Tri1p and Tri4p to toxisomes. Strain PH-1Tri1::GFP/Tri4::RFP was incubated in TBI medium for 36 h. Confocal bright field DIC (A); GFP (B); RFP (C); and GFP/RFP/DIC (D) overlay images of toxigenic cells are shown. The arrow in the GFP and RFP overlay image panel (E) highlights co-fluorescence of GFP and RFP within the periphery of a spherical organelle. A graph of signal intensity of GFP and RFP emission spectra (F) generated from a line bisecting the spherical organelle shows spatial overlap of the separate emission fluorescence. Scale bar = 10 µm.

GFP and RFP co-fluoresced under trichothecene biosynthesis inducing conditions in doubly tagged strains. These proteins were co-localized based on coincident traces of fluorescent intensity across circular structures (Figure 2). Additionally, z-stack imaging of cell fluorescence in induced cells was consistent with localization to the outer membrane of spherical organelles and, to a lesser extent, to the cellular endomembrane system (Video S1). Since Tri4p and Tri1p catalyze early and late steps in trichothecene synthesis in *Fusarium*, we infer these spherical organelles are a site of trichothecene biosynthesis and provisionally refer to them as “toxisomes.”.

### Vesicles Associated with Trichothecene Detoxification Interact with Toxisomes

**Figure 3:**
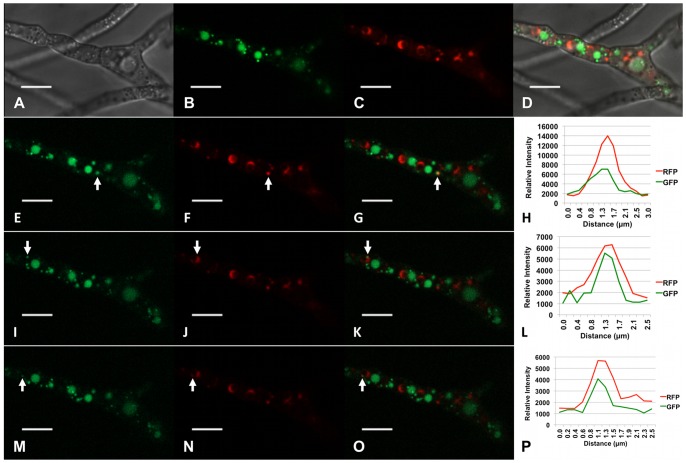
Interactions between toxisomes and Tri12p::GFP labeled vesicles. Co-localization of Tri4p::RFP labeled organelles and motile Tri12p::GFP labeled vesicles within strain PH-1Tri4::RFP/Tri12::GFPB under conditions where trichothecene biosynthesis occurs. Confocal bright field DIC (A); GFP (B, E, I, M); RFP (C, F, J, N); GFP/RFP/DIC overlay (D); and GFP/RFP overlay images (G, K, O) are shown. These images show strain PH-1Tri4::RFP/Tri12:GFPB co-expressing Tri4p::RFP and Tri12p::GFP fusion proteins 36 h after suspension in TBI medium. Signal intensity of GFP and RFP emission spectra (H, L, P) were generated from a line bisecting each organelle labeled (arrows) in panels E–G, I–K; and M–O. Panels E–G, I–K, and M–O show images of the same culture at different times. Images were captured from Video S2. Scale bar = 10 µm.

Previously we have shown a transporter protein encoded by a gene (*Tri12*) within the trichothecene biosynthetic gene cluster was responsible for tolerance of *F. graminearum* to growth inhibition under conditions that induce trichothecene biosynthesis [23]. In addition to being localized to the plasma membrane, Tri12p also localized to small (approximately 1.0 µm) motile vesicles and ultimately to larger, stationary vacuoles. To determine the relationship between motile vesicles, the vacuole and the toxisome, Tri12p was tagged with GFP in a Tri4::RFP genetic background. Under conditions that induce toxin biosynthesis, toxisomes, as well as Tri12p::GFP labeled vesicles and vacuoles are not randomly distributed within the cell (Figure 3). Rather, vesicles and vacuoles flank Tri4p::RFP labeled toxisomes in an orderly fashion; toxisomes always are arranged immediately adjacent to Tri12p::GFP labeled organelles. Moreover, motile vesicles appear to dynamically interact with toxisomes (Video S2). Fluorescence traces suggest transient co-localization of vesicles and toxisomes (Figure 3). We interpret these instances of transient co-localization as evidence for vesicular tethering or docking. However, during the interaction of vesicles and toxisomes, the organelles appear to remain separate as they eventually resolve as stationary toxisomes and motile vesicles. Following resolution of the vesicle from the toxisome, movement of the vesicle to Tri12p::GFP containing vacuoles and apparent fusion with vacuoles often occurs (Video S2).

### Interaction of Tri12p-labeled Vesicles and F-actin

**Figure 4:**
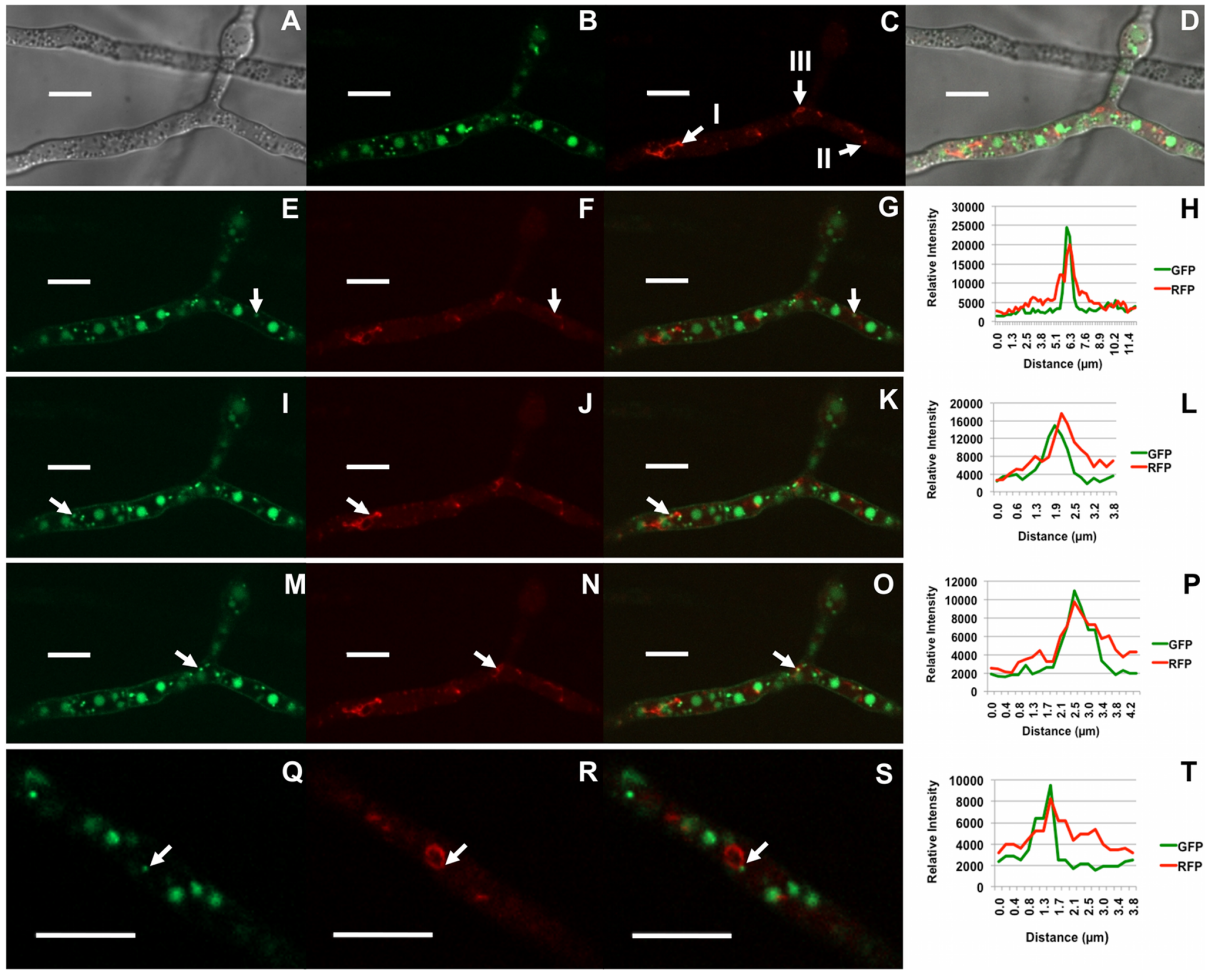
Close associations between Tri12p::GFP labeled vesicles and F-actin. Strain PH-1Tri12::GFP/Lifeact::RFP co-expressing Lifeact::RFP and Tri12p::GFP fusion proteins 36 h after suspension in TBI medium. Confocal bright field DIC (A), GFP (B, E, I, M, Q); RFP (C, F, J, N, R); DIC, GFP and RFP overlay (D); and GFP and RFP overlay images (G, K, O, S) are shown. Arrows in image C identify actin cables (I), an actin patch (II) and a lariat-like structure (III) composed of actin cables. Signal intensity of GFP and RFP emission spectra (H, L, P, T) were generated from a line bisecting the organelles labeled (arrows) in images E–G, I–K, M–O and Q–S. Panels E–G, I–K and M–O are images of the same culture at different times shown in Video S3. Panels Q–S are from a separate cell image captured from Video S4.

In a previous study [23], we demonstrated that movement of vesicles containing Tri12p::GFP was reversibly inhibited by the actin-depolymerizing macrolide, latrunculin A [24], suggesting vesicular movement relies on the actin cytoskeleton. To directly address this possibility, we visualized F-actin within toxigenic cells using a strain created to express the actin-binding Lifeact polypeptide [25] linked to RFP in a Tri12p::GFP genetic background. Lifeact::RFP fluorescence revealed F-actin structures similar to those described in other filamentous fungi [26], including actin patches and actin cables (Figure S1). Additionally, lariat-like structures also were detected in cells grown under conditions inducive and non-inducive to toxin biosynthesis (Figure 4). Interaction between Tri12p::GFP labeled vesicles and Lifeact::RFP labeled actin lariats, filaments and patches was inferred from close associations detected by transient co-fluorescence. Real time imaging of structures also was consistent with tethering and movement of Tri12p::GFP labeled vesicles along actin filaments and co-translocation of actin lariats led by these vesicles (Videos S3 and S4).

### Localization of Enzymes of Primary Metabolism and Secondary Metabolism in Toxigenic Cells

Farnesyl pyrophosphate is the starting substrate for trichothecene biosynthesis, and genes for all enzymes in the isoprenoid biosynthetic pathway from acetyl CoA to farnesyl pyrophosphate (in addition to all genes of the trichothecene biosynthetic pathway) are positively regulated by the transcription factor Tri6p [22]. To determine the location of an enzymatic component of the isoprenoid pathway in toxigenic cells, the enzyme 3-hydroxy-3-methyl-glutaryl (HMG) CoA reductase (Hmr1p) was tagged with GFP in *F. graminearum*. Hmr1p has eight predicted transmembrane domains at the N-terminus [27].

**Figure 5:**
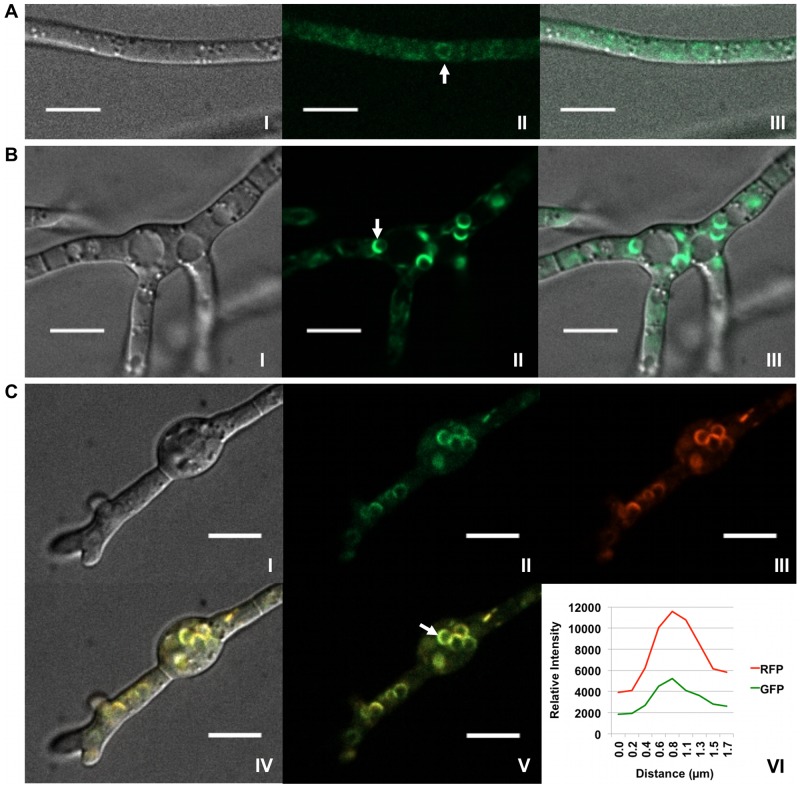
Re-patterning of Hmr1p fluorescence in toxin induced cells. (A) Localization of Hmr1p::GFP in strain PH-1Hmr1::GFP 36 h after suspension in MM where trichothecene biosynthesis does not occur. Confocal bright field DIC (I); GFP (II); and DIC and GFP (III) overlay images are shown. Hmr1p::GFP fluorescence corresponds mostly to diffuse membranous structures within the cell but occasionally to spherical bodies (arrow). (B) Localization of Hmr1p::GFP in strain PH-1Hmr1::GFP 36 h after suspension in TBI medium. Confocal bright field DIC (I); GFP (II); DIC and GFP (III) overlay images show a shift in localization of Hmr1p::GFP (arrow) primarily to spherical organelles. (C) Co-localization of Hmr1p::GFP and Tri4p::RFP in strain PH-1Hmr1::GFP/Tri4::RFP 36 h after suspension in TBI medium. Confocal bright field DIC (I); GFP (II); RFP (III); and GFP/RFP/DIC (IV) overlay images are shown. Co-fluorescence of GFP and RFP (V) within the periphery of a spherical organelle (arrow) and signal intensity of GFP and RFP emission spectra (VI) generated from a line bisecting the spherical organelle show spatial overlap of the separate emission fluorescence. Scale bar = 10 µm.

During growth in minimal medium (MM), a condition under which trichothecene biosynthesis is not induced, strains expressing Hmr1p::GFP show only weak cytoplasmic fluorescence (Figure 5a), consistent with previously reported localization of the enzyme to the cellular endomembrane system [27]. A few spherical structures approximately 3 µm in diameter also fluoresce weakly in MM. However, during growth in TBI medium, fluorescence from Hmr1p::GFP is increasingly localized in spherical structures after 24 to 36 hours of incubation (Figure 5b, Figure S2, Video S5), with the greatest fluorescent signal visible at the periphery of toxisomes at 36 hours, as judged by co-localized fluorescence of Tri4p::RFP in doubly tagged strains (Figure 5c).

As peroxisomes have been implicated as a site of isoprenoid and secondary metabolite synthesis in filamentous fungi, we sought to determine if peroxisomes may be involved in the biogenesis of toxisomes in *F. graminearum*. To do this, the membrane associated peroxisomal assembly protein peroxin-3 (Pex3p) was tagged with GFP in a Tri4::RFP strain.

When grown under trichothecene biosynthesis inducing conditions, doubly tagged strains showed different localization patterns of the two fluorescent proteins. Pex3p::GFP localizes to distinct spherical or fused doubly spherical organelles, the peroxisomes, which are approximately 1 µm in diameter and are dispersed throughout the mycelium (Figure S3). After growing for 30 hours in TBI medium, these organelles may be stationary or may move (approximately 5 µm/sec), sometimes over long distances within the cell. Peroxisomes revealed by Pex3p::GFP are distinct from the toxisomes revealed by Tri4p::RFP, although co-localization of fluorescence from these proteins was occasionally observed (Video S6).

## Discussion

This is the first publication to describe the subcellular localization of enzymes involved in the biosynthesis of trichothecene mycotoxins. Two enzymes in the reaction pathway, FgTri1p and FgTri4p, were localized to spherical organelles approximately 3 to 4 µm in diameter that are the presumed site of trichothecene biosynthesis, hence the provisional name toxisome. We infer that trichothecene reaction products and intermediates are within the lumen of toxisomes, as the membrane anchoring domains of each cytochrome P-450 are near the predicted external N-terminus of the protein, while the heme binding domains, and thus the enzyme reaction sites, are near the C terminus predicted to be internally localized.

Targeting enzymes of a secondary metabolic pathway to a common organelle may be advantageous to cells for several reasons. First, precursors and reaction intermediates are kept within a relatively small volume, promoting their proximity to successive enzymes and likely enhancing efficiency of flux of intermediates through the pathway. Secondly, several steps in trichothecene biosynthesis require oxygenations catalyzed by at least three separate cytochrome P-450 enzymes: Tri1p, Tri11p and Tri4p. In order to generate the electrochemical potential for reduction of molecular oxygen, a short electron transport chain is required and likely includes the shared NADPH cytochrome P450 reductase FGSG_09786, [a protein, like the cytochrome P450s, also positively regulated (P = 0.01) by the transcription factor Tri6p [22]]. Although we have not yet demonstrated the presence of the conserved NADPH cytochrome P450 reductase in the toxisome, we predict its position there would generate the membrane potential driving each oxygenation step in the trichothecene pathway. Finally, any trichothecene reaction intermediates containing the C12–C13 epoxide moiety would likely be toxic. Thus, sequestration of these intermediates within the toxisome may protect the cell from their potentially damaging effects.

It remains to be determined whether the toxisome is a unique structure specific for trichothecene biosynthesis or whether it may be a conserved structural element involved in synthesis of other secondary metabolites in *F. graminearum* or other fungi. Geranylgeranyl diphosphate synthase 2 (GGS2), involved in the first committed step in biosynthesis of the isoprenoid gibberellins in the fungus *F. fujikuroi*, has a punctate distribution in cells distinct from the peroxisome [28] suggesting it may also be localized to an organelle similar to a toxisome. However, unlike the situation described here, HMG CoA reductase in *F. fujikuroi* does not co-localize with GGS2.

Toxisomes are clearly distinct from previously described Tri12p containing organelles [23]. The spatial arrangement of toxisomes and Tri12p containing organelles is not random and, indeed, toxisomes are invariably flanked by organelles containing Tri12p. Previously, we observed that Tri12p was involved in trichothecene tolerance in *F. graminearum* and showed localization of the protein to the plasma membrane (PM), the vacuole and to small (<1 µm) motile, and at times oscillating, vesicles. Formerly, we emphasized the position of Tri12p in the PM as being central to its function. However, in the current investigation, after viewing the juxtaposition of Tri12p containing vacuoles with toxisomes, the interaction between Tri12p vesicles and toxisomes, and the apparent docking and oscillating movement between the toxisome and the vacuole, we propose a broader role for Tri12p.

**Figure 6:**
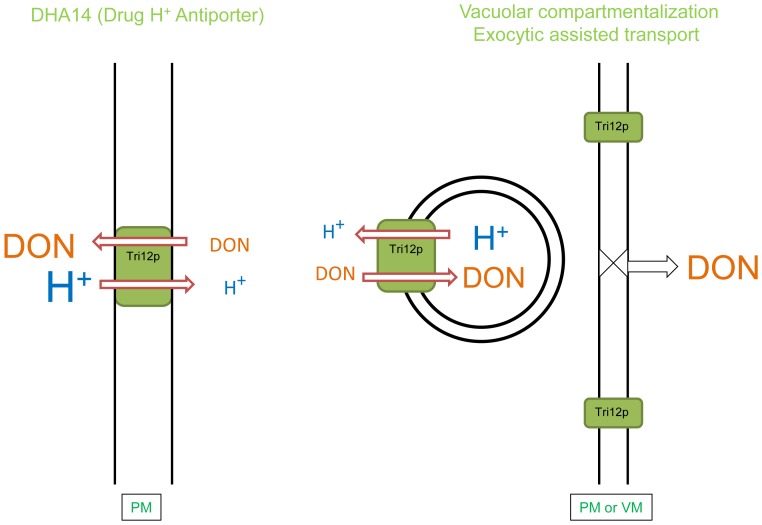
Models of Tri12p facilitated trichothecene export. (Left) Based on predicted amino acid sequence similarity, Tri12p corresponds to a DHA14 (Drug/H^+^ antiporter with 14 membrane spanning domains) major facilitator transporter. Localization of Tri12p in the plasma membrane (PM) may directly lead to trichothecene (DON) export. (Right) Localization of Tri12p in the membrane of small motile vesicles may lead to accumulation of DON within. Upon fusion with the PM or the vacuolar membrane (VM), concentrated DON may be, respectively, eliminated by exocytosis or compartmentalized within the vacuole.

According to our model, the protein may function in two ways (Figure 6): First, it may serve as a DHA14 drug antiporter [29], as predicted by its conserved structure, and, by virtue of its position in the PM, may move trichothecenes across the PM to outside the cell. This is in agreement with work on the *F. sporotrichioides* (Fs)Tri12 transporter showing that FsTri12p expressed in yeast facilitates trichothecene export from cells [30]. Secondly, we propose that Tri12p also may serve to accumulate trichothecenes within vesicles and the vacuole (Figure 6). Because of the predicted direction of Tri12p within the membrane, vesicles may deliver the protein to the PM and, in doing so, also assist in export of concentrated trichothecene from the cell. Likewise, vesicular trafficking of Tri12p to the vacuole may include the transport of trichothecenes to this organelle. The vacuole may sequester toxins from sensitive organelles such as ribosomes and mitochondria [17], [31].

The motility of vesicles containing Tri12p was reversibly inhibited by latrunculin A, indicating that movement was dependent upon filamentous actin [23]. To further test involvement of F-actin in vesicular trafficking, the protein was directly imaged using the Lifeact polypeptide derived from actin binding protein Abp140 [32], [25] fluorescently tagged with RFP. Interactions between F-actin and Tri12p were inferred by Lifeact::RFP and Tri12p::GFP co-fluorescence. Due to its low affinity and non-covalent binding [26], Lifeact::RFP likely did not allow for visualization of all F-actin containing elements within each cell. Nevertheless, F-actin cables, patches and lariats revealed by Lifeact in *F. graminearum* imply remarkable interactions. In certain instances, movement of Tri12p::GFP labeled vesicles appears to occur bi-directionally along F-actin cables (Video S3). These vesicles also interact with actin rings, at times appearing to be an attachment point for translocation of the lariats within the cell (Video S4).

Hmr1p, an enzyme involved in the primary metabolic pathway for isoprenoid biosynthesis, localized in the ER during growth in MM as previously noted for *F. graminearum* [27]. Additionally, under these trichothecene biosynthesis non-inducing conditions, GFP-tagged Hmr1p also localizes to the periphery of spherical organelles roughly the size of toxisomes (Figure 5a). Upon the shift of *F. graminearum* cells to trichothecene biosynthesis inducing conditions, the number and intensity of fluorescence of spherical organelles increases. At 24 hours, GFP-tagged Hmr1p is primarily seen within spherical structures (Figure 5b) within ovoid toxigenic cells that co-localize with Tri4p::RFP in doubly tagged strains. By 36 hours, the most intense concentration of Hmr1p occurs within toxisomes, as judged by co-fluorescence of Hmr1p::GFP and Tri4p::RFP in doubly tagged strains (Figure 5c). Based on these observations, we speculate that toxisomes may develop from organelles already formed within non-toxigenic cells. A shift to conditions that induce trichothecene biosynthesis may result in the proliferation of these organelles and the targeting of trichothecene biosynthetic enzymes to these locations. We are currently investigating the possibility that toxisomes may develop from pre-existing organelles that function in primary metabolism, for example, those involved in ergosterol biosynthesis. Regardless, ergosterol and other isoprenoid derived primary metabolites still must be made within toxigenic cells. It remains to be determined whether the entire mevalonate pathway, or just the portion channeled toward trichothecene biosynthesis, localizes to the toxisome. Indeed, the exact relationship between toxisomes, Tri12p vesicles and various components of vesicular trafficking in non-toxigenic cells remains to be thoroughly examined.

For synthesis of the polyketide aflatoxin in *Aspergillus* species, Roze *et al*., (2011) have proposed that peroxisomes are a source of acetyl CoA, the basic biochemical precursor for polyketides, as well as the site of early steps in aflatoxin biosynthesis [33]. Since trichothecenes also are ultimately derived from acetyl CoA, we tested whether toxisomes in *F. graminearum* may arise from peroxisomes. Strains were created that were GFP-tagged for Pex3p, which is targeted to the peroxisomal membrane, and RFP-tagged for Tri4p, which is targeted to the toxisome. The two fluorescently labeled proteins within the same cell revealed two distinct localization patterns during all developmental phases examined, with Tri4p::RFP targeted to the spherical 3–4 µm toxisome and Pex3p::GFP targeted to the much smaller, and developmentally distinct 0.5–1 µm peroxisome. It is interesting to note that in toxigenic cells expressing Tri4p::RFP, peroxisomes are often non-motile, while in non-toxigenic cells, whether in MM or prior to induction in TBI medium, peroxisomes are highly mobile but may exhibit transient co-localization (Video S6).

Our model for compartmentalization of trichothecene biosynthesis resembles one proposed for synthesis and export of aflatoxins in the fungus *Aspergillus* [33]. Each model suggests that toxin synthesis occurs within a specialized vesicular organelle; either the aflatoxisome proposed by Chandra *et al*. [9], or the toxisome proposed here. Each model also proposes that toxin export may occur by transmembrane transporter proteins located in the plasma membrane or, additionally, may occur by exocytosis [16], [23]. Beyond these suggestions the models diverge. Aflatoxisomes have been proposed to develop by budding of peroxisomes, in which early biosynthetic steps occur and which later fuse either with vesicles targeted to the vacuole or with ER-derived secretory vesicles destined for exocytosis. The vacuole directed vesicles and secretory vesicles are proposed to contain enzymes catalyzing the later steps in aflatoxin synthesis [33].

**Figure 7:**
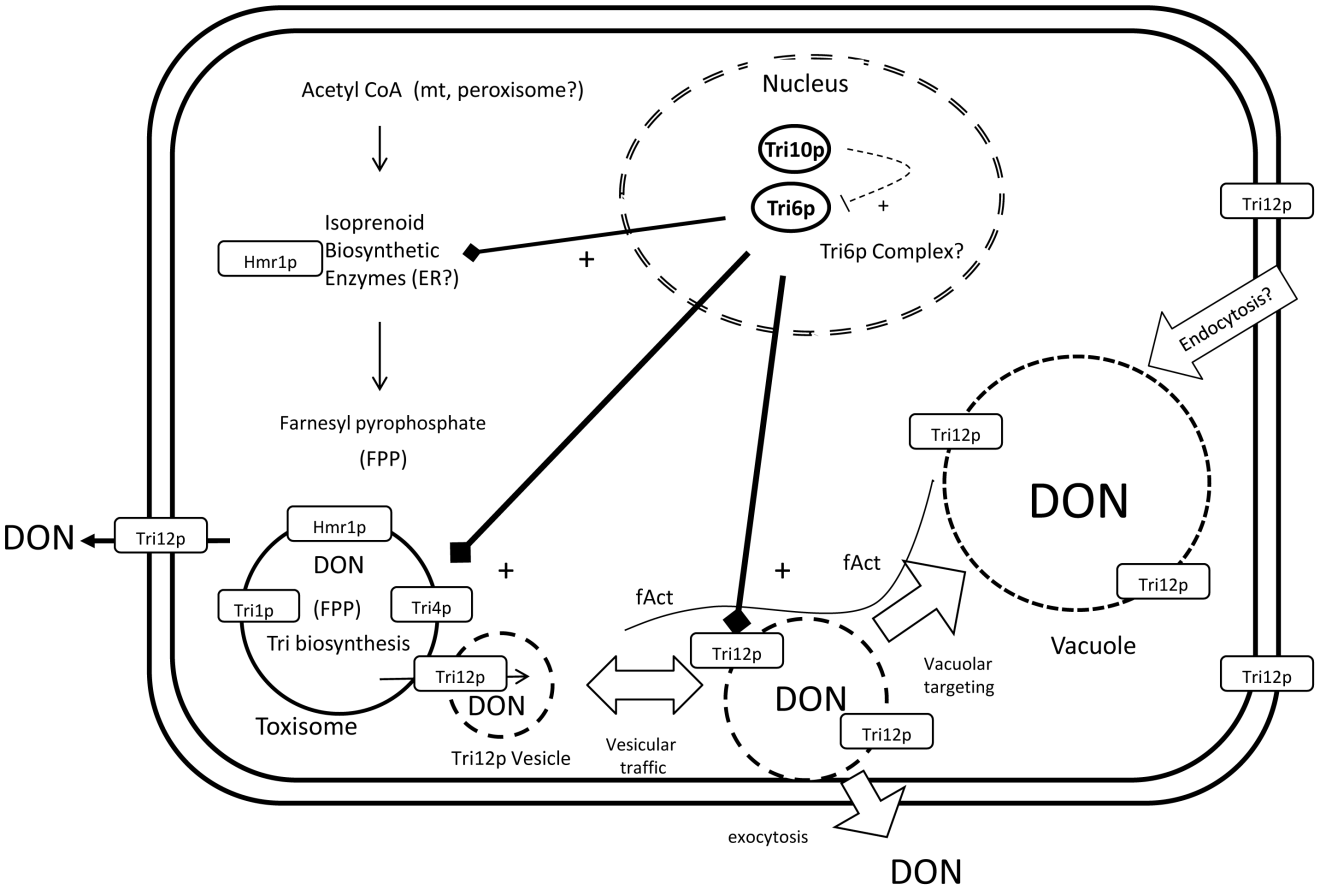
Model of trichothecene biosynthesis and export in *F.graminearum*. Within the nucleus, transcription factors Tri6p and Tri10p positively regulate transcription of genes involved in trichothecene (DON) biosynthesis and tolerance [22]. The genes for enzymes in the isoprenoid biosynthesis pathway, including HMG CoA reductase (*Hmr1*), are constitutively expressed but are up-regulated during DON synthesis. DON biosynthesis induction leads to a shift in Hmr1p targeting from the ER to the toxisome. The vesicular toxisome is the site of trichothecene biosynthetic enzymes Tri1p and Tri4p, proteins that also are up-regulated under the control of Tri10p and Tri6p during DON biosynthesis. Also regulated by these transcription factors is the DHA14 protein Tri12p that confers a level of tolerance to DON [23]. Motility of Tri12p::GFP labeled vesicles appears to be dependent upon F-actin and results in fusion with the vacuole and the plasma membrane [23]. DON synthesis therefore may be sequestered within the toxisome and export of toxic products may be facilitated by trafficking of Tri12p::GFP labeled vesicles.

For trichothecene synthesis, we propose (Figure 7) toxisomes may develop directly from the cellular ER - Golgi apparatus and may contain not only enzymes specific for trichothecene biosynthesis, but also may contain enzymes in the primary metabolic pathway for isoprenoid synthesis. Unlike aflatoxisomes, trichothecene toxisomes have not been observed to fuse with the plasma membrane. Rather, our model proposes toxin export may occur by the intervention of Tri12p containing vesicles. These vesicles may dock with toxisomes, accumulate or transfer trichothecenes to the vacuole, or export trichothecenes by fusion with the plasma membrane. The model we present for trichothecene synthesis neither supports nor refutes any element of the aflatoxisome model. Each fungus may have independently evolved mechanisms for toxin sequestration and export utilizing conserved elements of its own cellular biosynthetic machinery.

Several questions need to be addressed to strengthen our understanding of the compartmentalization of secondary metabolism and toxigenesis in *F. graminearum* and filamentous fungi in general. For example, do Tri12p containing vesicles incorporate components of common vesicular trafficking pathways and employ elements of the conserved exocytosis machinery of the cell? What are the signals and mechanisms by which the trichothecene biosynthetic proteins are targeted to the toxisome? Are other plant-induced *Fusarium* secondary metabolites (e.g. zearelanone) produced within toxisomes? Are structures comparable to the toxisome involved in synthesis of secondary metabolites in other filamentous fungi? These questions will motivate our continued work toward a clearer understanding of how filamentous fungi manufacture and export their amazing diversity of small bioactive molecules.

## Materials and Methods

### Strains and Culture Conditions

*F. graminearum* wild type strain PH-1 (NRRL 31084) and all mutants were cultured at 25°C in liquid carboxymethylcellulose (CMC) medium [low viscosity CMC (Sigma-Aldrich, St. Louis), 15.0 g; NH_4_NO_3_, 1.0 g; KH_2_PO_4_, 1.0 g; MgSO_4_-7H_2_O, 0.5 g; yeast extract (BD, Franklin Lakes, NJ) 1.0 g] for five days. Spores were harvested by low speed centrifugation and washed twice with sterile deionized distilled H_2_O. Spore concentrations were determined using a hemacytometer. Minimal medium (MM) was prepared as previously described [23]. Liquid trichothecene biosynthesis induction (TBI) medium was prepared as previously described [34] with minor modification. Trace element solution for Hmr1 and Pex3 experiments was supplemented with 1.0 g Fe(NH_4_)_2_(SO_4_)_2_· 6H_2_O per 95 ml.

### Protoplasts and Transformation

Culturing of source tissue, protoplast preparation and fungal transformation were performed as described previously [23], [35]. Protoplasts were isolated using 500 mg driselase (Sigma-Aldrich, St. Louis) and 200 mg lysing enzymes from *Trichoderma harzianum* (Sigma-Aldrich, St. Louis) in 20 ml 1.2 M KCl, incubated at 30°C with shaking at 80 rpm. Following protoplast formation, the suspension was filtered through two sheets of miracloth (EMD Millipore, Billerica, MA). The filtrate was centrifuged and the protoplast pellet washed three times with 1.2 M KCl. Final resuspension was to 10^7^–10^8^ protoplasts ml^−1^ in 93% 1.2 M STC (1.2 M sorbitol, 10 mM Tris-HCl (pH 8.0), 50 mM CaCl_2_), 7% DMSO. Protoplasts were distributed into 200 µl aliquots which were stored at −80°C.

Transformation of protoplasts was performed by adding 1–10 µg of transforming DNA to a 200 µl protoplast aliquot. Following 20 minutes incubation on ice, 1 ml 40% PEG 8000 (Sigma-Aldrich, St. Louis) in 1.2 M STC was added and the reaction incubated an additional 20 minutes at room temperature. The transformation reaction was transferred to 5 ml liquid TB3 (0.3% yeast extract, 0.3% casamino acids, 0.6 M sucrose) for regeneration, and cultures incubated for 16 h at 25°C with shaking at 150 rpm. Regenerated protoplasts were collected by centrifugation and resuspended in 1 ml 1.2 M STC. One third of the resuspension (approximately 350 µl) was added to 5 ml TB3 containing 0.7% low melting point agarose (Lonza, Allendale, NJ), and overlaid onto 7.5 ml solid TB3 (liquid TB3 with 0.7% low melting temperature agarose) containing 150 µg ml^−1^ hygromycin B (Roche Applied Science, Indianapolis, IN). Three selection plates were prepared for each transformation reaction. Plates were incubated for 16 hours at 25°C in the dark and then overlaid with 7.5 ml solid TB3 containing 250 µg ml^−1^ hygromycin B and incubated at 25°C in the dark until resistant colonies were visible (approximately 7 days). Resistant colonies were isolated onto V8 medium containing 250 µg ml^−1^ hygromycin B and incubated for 7 days at 25°C. Conidia from the V8 plates were then used for isolating monoconidial strains of each putative transformant. Monoconidial strains were used for further characterization. For selection with nourseothricin (United States Biological, Swampscott, MA), regenerated protoplasts were overlaid onto solid TB3 containing 25 µg ml^−1^ nourseothricin, with the second TB3 overlay containing 50 µg ml^−1^ nourseothricin, and resistant colonies isolated onto potato dextrose agar (PDA) or V8 juice agar (BD, Franklin Lakes, NJ) containing 50 µg ml^−1^ nourseothricin_._

### DNA Extraction and Southern Blotting

Culturing of source tissue and DNA extraction were performed as described previously [23]. Genomic DNA (20 µg/sample) was digested with XbaI, XmnI, or BglII. DNA probes were used to detect the presence of *GFP*, *RFP*, *Tri1, Tri4*, and *Tri12* in the appropriate fungal strains via Southern hybridization. DNA oligonucleotides listed in Table S1 were used to synthesize these probes. Southern hybridization, probe labeling and detection were performed as described previously [36], [23].

### GFP and RFP Tagging

A fusion PCR-based method [37] was used to synthesize the constructs for generating full-length proteins tagged with GFP or TagRFP-T (hereafter called RFP). Strains created were PH-1Tri1::GFP, PH-1Tri4::RFP, PH-1Tri1::GFP/Tri4::RFP, PH-1Tri12::GFP/Tri4::RFP, PH-1Tri12::GFP/Lifeact::RFP, PH-1Hmr1::GFP/Tri4::RFP and PH-1Pex3::GFP/Tri4::RFP. The *Neurospora* knock-in vector [38] pGFP::hph::loxP (GenBank: FJ457011.1) was used as a template for the synthesis of the *GFP::hph* portion of the fusion constructs. The pAL12-Lifeact vector (Fungal Genetics Stock Center, Kansas City) was used as a template for the synthesis of the construct used to create strain PH-1Lifeact::RFP and the *RFP::nat1* portion of fusion constructs. Oligonucleotides used to amplify the upstream and downstream regions flanking the *Tri1, Tri4*, *Pex3*, and *Hmr1* stop codons and *GFP::hph* and *RFP::nat1* and Lifeact cassette constructs are listed in Table S1. Hygromycin and nourseothricin resistant transformants were isolated with V8 juice agar supplemented with 250 mg ml^−1^ hygromycin B and PDA or V8 juice agar containing 50 mg ml^−1^ nourseothricin. Integration of GFP or RFP tagging constructs was confirmed via Southern hybridization or PCR amplification of DNA with gene specific oligonucleotides (Figures S4–S6, Table S1).

### Protein Extraction, Western Blotting, Trichothecene Accumulation Assay

Western blotting was used to confirm the presence of GFP and RFP tagged fusion proteins in cellular extracts from strains PH-1Tri1::GFP, PH-1Tri4::RFP, PH-1Tri1::GFP/Tri4::RFP, PH-1Tri12::GFP/Tri4::RFP, and PH-1Tri12::GFP/Lifeact::RFP (Figure S7–S9). Culture conditions, sampling, handling of mycelia, protein extraction and Western blotting of strains were performed as described previously [23]. Western blots were probed sequentially with primary goat anti-GFP (Santa Cruz Biotechnology, Santa Cruz, CA) or rabbit anti-RFP antibodies (Life Technologies, Carlsbad, CA) and secondary rabbit anti-goat or goat anti-rabbit HRP antibodies (Santa Cruz Biotechnology, Santa Cruz, CA). The molecular weights of the Tri1p::GFP fusion protein, free GFP, Tri4p::RFP fusion protein and free RFP were similar to the calculated expected masses of these proteins when compared to size standards via SDS -PAGE. Samples of filtered TBI culture medium were freeze-dried and analyzed for the presence of DON and 15ADON as previously described [23] [39]. All strains containing fluorescent protein fusions were able to produce trichothecenes (≥2 ppm 15ADON) when tested at 36 or 48 h.

### Microscopy

To observe the expression of fluorescent proteins *in vivo*, conidia were suspended in 50 ml TBI cultures at a final concentration of 10^4^ conidia ml^−1^ and grown at 28°C on an orbital shaker at 150 rpm in total darkness. A Zeiss Cell Observer SD spinning disk confocal microscope (Carl Zeiss AG, Oberkochen, Germany) was used to image wet mounts of live cells. ZEN lite 2011 software was used for image generation and analysis (Carl Zeiss AG). Imaris 7.6.0 software (Bitplane Inc, South Windsor, CT) was used for 3D renderings shown in Video S1 and S5.

## Supporting Information

Figure S1 **F-actin bound by Lifeact::RFP.** Confocal bright field DIC (A), RFP (B), RFP and DIC overlay (C) images of Lifeact::RFP cells captured after 36 h of incubation in MM at 28°C in total darkness are shown. Actin cables (I); patches (II); and lariat-like structures (III) composed of actin cables are present. Scale bar = 10 µm.
(TIF)

Figure S2 **Visualization of Hmr1p and Tri4p under trichothecene biosynthesis inducing conditions.** Expression of Hmr1p::GFP and Tri4p::RFP in strain PH-1Hmr1::GFP/Tri4::RFP under conditions where trichothecene biosynthesis occurs. Confocal bright field DIC (A); GFP (B); RFP (C); GFP and RFP overlay (D); and GFP, RFP and DIC overlay (E) images are shown of strain PH-1Hmr1::GFP/Tri4::RFP in TBI medium after 24 h incubation at 28°C in total darkness. Hmr1p::GFP expression is widespread among cells while Tri4p::RFP expression is limited. Scale bar = 10 µm.
(TIF)

Figure S3 **Visualization of Pex3p and Tri4p under trichothecene biosynthesis inducing conditions.** Co-expression of Pex3p::GFP and Tri4p::RFP in strain PH-1Pex3::GFP/Tri4::RFP under conditions where trichothecene biosynthesis occurs. Confocal DIC (A); GFP (B); RFP (C); GFP and RFP overlay (D); and GFP, RFP and DIC overlay (E) images are shown of the strain in TBI medium after 24 h incubation at 28°C in total darkness. Pex3p::GFP and Tri4p::RFP localize exclusively to peroxisomes and toxisomes, respectively. Scale bar = 10 µm.
(TIF)

Figure S4 **Southern hybridization of genomic DNA from strains expressing Tri1p::GFP.** XbaI restriction enzyme fragment cut sites in PH-1 (A) and Tri1p::eGFP (B) transformants and the expected sizes of fragments targeted by hybridization probes are shown. Hybridization of probes for *Tri1* (C) and *GFP* (D) to XbaI digested genomic DNA from PH-1 (I); PH-1Tri1::GFPA; (II) PH-1Tri1::GFPB (III); and PH-1Tri1::GFP/Tri4::RFP (IV) is shown. These results demonstrate the presence of single copies of *Tri1* in all strains and single copies of GFP in the transformed strains. The relative sizes of the labeled fragments are consistent with expected digestion patterns.
(TIF)

Figure S5 **Southern hybridization of genomic DNA from strains expressing Tri4p::RFP.** BglII restriction enzyme cut sites in PH-1 (A) and Tri4::RFP (B) transformants and the expected sizes of fragments targeted by hybridization probes are shown. Hybridization of probes for *Tri4* (C) and *RFP* (D) to BglII digested genomic DNA from PH-1 (I); PH-1Tri4::RFPA (II); PH-1Tri4::RFPB; and III) PH-1Tri1::GFP/Tri4::RFP (IV) are shown. These results demonstrate the presence of single copies of *Tri4* in all strains and single copies of *RFP* in the transformed strains. The relative sizes of the labeled fragments are consistent with expected digestion patterns.
(TIF)

Figure S6 **Southern hybridization of genomic DNA from strains expressing Tri12p::GFP and Tri4p::RFP or Tri12p::GFP and Lifeact::RFP.** XcmI restriction enzyme cut sites in PH-1 (A) and Tri12p::GFP expressing strains (B) and the expected sizes of fragments targeted by hybridization probes are shown. Hybridization of probes for *Tri12* (C), *GFP* (D) and *RFP* (E) to Xcm1 digested genomic DNA from PH-1Tri12::GFP/Tri4::RFP-A (I); PH-1Tri12::GFP/Tri4::RFPB (II); PH-1Tri12::GFP/Lifeact::RFPA (III); PH-1Tri12::GFP/Lifeact::RFPB (IV); PH-1 (V); and PH-1Lifeact::RFP (VI) is shown. These results demonstrate the presence of single copies of *Tri12* in all strains; single copies of *GFP* in all strains except PH-1 and PH-1Lifeact::RFP; and single copies *RFP* in all strains except PH-1. The GFP probe hybridized to fragments containing the coding region of *RFP* as demonstrated by the RFP probe hybridization pattern in panel C. The relative sizes of the labeled fragments are consistent with expected digestion patterns.
(TIF)

Figure S7 **Western blots for Tri1p::GFP.** (A) A model of the Tri1p::GFP fusion protein (I); the approximate mass of the full-length fusion protein (87.1 kD) (II); and the approximate masses of untagged Tri4p (59.2 kDa) and GFP (27.9 kDa) (III). (B) Western blots of protein extracts from PH-1Tri1::GFPA (I) and PH-1Tri1::GFPB (II) cultures obtained at 24 (a), 30 (b), 36 (c) and 48 (d) h after inoculation of TBI medium confirm the presence of full-length Tri1p::GFP (i) and GFP (ii) after 36 h. The approximate masses of these proteins are consistent with molecular weight estimates.
(TIF)

Figure S8 **Western blots for Tri4p::RFP.** (A) A model of the Tri4p::RFP fusion protein (I); the approximate mass of the full-length fusion protein (87.4 kDa) (II); and the approximate masses of untagged Tri4p (59.2 kDa) and RFP (28.2 kDa) (III). (B) Western blots of protein extracts from PH-1Tri4::RFPA (I) and PH-1Tri4::RFPB (II) cultures obtained at 24 (a), 30 (b), 36 (c) and 48 (d) h after inoculation of TBI medium confirm the presence of full-length Tri4p::RFP (i) and RFP (ii) after 36 h. The approximate masses of these proteins are consistent with molecular weight estimates. A third protein detected by the anti-RFP antibody is likely an intermediate product resulting from the partial digestion of the Tri4p::RFP fusion protein.
(TIF)

Figure S9 **Western blots for Tri1p::GFP and Tri4p::RFP.** Western blots of protein extracts from tissue samples obtained from a TBI culture of PH-1Tri1::GFP/Tri4::RFP. Protein extracts from a PH-1Tri1::GFP/Tri4::RFP culture were probed with anti-GFP (A) or anti-RFP (B) antibodies. Samples obtained at 24 (I), 30 (II), 36 (III) and 48 (IV) h after inoculation of TBI medium confirm the presence of full-length fusion proteins (a) and GFP or RFP (b) after 36 h.
(TIF)

Table S1 Oligonucleotides used for the synthesis of tagging constructs, Southern blots and PCR confirmation of GFP and RFP tagged strains.
(DOCX)

Video S1 **Co-localization of Tri1p and Tri4p.** A three-dimensional rendering of a z-stack image capture series obtained for strain PH-1Tri1::GFP/Tri4::RFP during induction of trichothecene biosynthesis. This rendering of GFP and RFP overlay images of cells in TBI medium after 36 h of incubation at 28°C in total darkness shows co-localization of Tri1p::GFP and Tri4p::RFP in multiple cells. Scale bar = 5 µm.
(MP4)

Video S2 **Interaction of Tri12p::GFP labeled vesicles and toxisomes.** Time-lapse imaging of strain PH-1Tri4::RFP/Tri12::GFPB during induction of trichothecene biosynthesis. Co-fluorescence of a motile Tri12p::GFP labeled vesicle occurs with a toxisome labeled with Tri4p::RFP (A) when grown in TBI medium for 36 h. Movement of a Tri12p::GFP labeled vesicle to a vacuole with apparent fusion (B). Other instances of Tri12p::GFP labeled vesicles interacting with Tri4p::RFP labeled toxisomes were also observed (C, D). Time codes in the upper right- and lower left-hand corners of the video indicate the relative times of image captures (in seconds) for the GFP and RFP channels, respectively. Scale bar = 10 µm.
(MP4)

Video S3 **Interaction of Tri12p::GFP labeled vesicles and F-actin.** Time-lapse imaging of strain PH-1Tri12::GFP/Lifeact::RFPA during induction of trichothecene biosynthesis. Motile Tri12p::GFP labeled organelles (A, B, C) are often in close association with actin cables after 36 h in TBI medium. Time codes in the upper right- and lower left-hand corners of the video indicate the relative times of image captures (in seconds) for the GFP and RFP channels, respectively. Scale bar = 10 µm.
(MP4)

Video S4 **Interaction of a Tri12p::GFP labeled vesicle and an F-actin lariat.** Additional time-lapse imaging of strain PH-1Tri12::GFP/Lifeact::RFPA during induction of trichothecene biosynthesis. The video shows co-translocation (arrow) of single motile Tri12p::GFP labeled vesicle and a lariat-like structure labeled with Lifeact::RFP after 36 h in TBI medium. Time codes in the upper right- and lower left-hand corners of the video indicate the relative times of image captures (in seconds) for the GFP and RFP channels, respectively. Scale bar = 10 µm.
(MP4)

Video S5 **Co-localization of Hmr1p and Tri4p.** Three-dimensional rendering of a z-stack image capture series obtained for strain PH-1Hmr1::GFP/Tri4::RFP during induction of trichothecene biosynthesis. This rendering of GFP and RFP overlay images of cells after 36 h in TBI medium shows co-localization of Hmr1p::GFP and Tri4p::RFP in multiple cells. Scale bar = 5 µm.
(MP4)

Video S6 **Toxisomes and peroxisomes imaged during induction of trichothecene biosynthesis.** Time-lapse imaging of strain PH-1Pex3::GFP/Tri4::RFP during induction of trichothecene biosynthesis. Pex3p::GFP labeled peroxisomes exhibit motility relative to stationary Tri4p::RFP labeled toxisomes after 36 h incubation in TBI medium. Time codes in the upper right- and lower left-hand corners of the video indicate the relative times of image captures (in seconds) for the GFP and RFP channels, respectively. Scale bar = 10 µm.
(MP4)

## References

[1] Bennett JW, Klich M (2003) Mycotoxins. Clin Microbiol Rev 16: 497–516 doi:10.1128/CMR.16.3.497-516.2003. 12857779 10.1128/CMR.16.3.497-516.2003 PMC164220

[2] Stadler M, Keller NP (2008) Paradigm shifts in fungal secondary metabolite research. Mycological Research 112: 127–130 doi:10.1016/j.mycres.2007.12.002. 18319144 10.1016/j.mycres.2007.12.002

[3] Hoffmeister D, Keller NP (2007) Natural products of filamentous fungi: enzymes, genes, and their regulation. Nat Prod Rep 24: 393–416 doi:10.1039/b603084j. 17390002 10.1039/b603084j

[4] Nierman WC, Pain A, Anderson MJ, Wortman JR, Kim HS, et al (2005) Genomic sequence of the pathogenic and allergenic filamentous fungus *Aspergillus fumigatus* . Nature 438: 1151–1156 doi:10.1038/nature04332. 16372009 10.1038/nature04332

[5] Cuomo CA, Güldener U, Xu J-R, Trail F, Turgeon BG, et al (2007) The *Fusarium graminearum* genome reveals a link between localized polymorphism and pathogen specialization. Science 317: 1400–1402 doi:10.1126/science.1143708. 17823352 10.1126/science.1143708

[6] Müller WH, Bovenberg RAL, Groothuis MH, Kattevilder F, Smaal EB, et al (1992) Involvement of microbodies in penicillin biosynthesis. Biochimica et Biophysica Acta (BBA) - General Subjects 1116: 210–213 doi:10.1016/0304-4165(92)90118-E. 1581347 10.1016/0304-4165(92)90118-e

[7] Lendenfeld T, Ghali D, Wolschek M, Kubicek-Pranz EM, Kubicek CP (1993) Subcellular compartmentation of penicillin biosynthesis in *Penicillium chrysogenum*. The amino acid precursors are derived from the vacuole. J Biol Chem 268: 665–671. 8416970

[8] Spröte P, Brakhage AA, Hynes MJ (2009) Contribution of peroxisomes to penicillin biosynthesis in *Aspergillus nidulans* . Eukaryotic Cell 8: 421–423 doi:10.1128/EC.00374-08. 19151327 10.1128/EC.00374-08 PMC2653248

[9] Chanda A, Roze LV, Kang S, Artymovich KA, Hicks GR, et al (2009) A key role for vesicles in fungal secondary metabolism. Proc Natl Acad Sci U S A 106: 19533–19538 doi:10.1073/pnas.0907416106. 19889978 10.1073/pnas.0907416106 PMC2773199

[10] Teijeira F, Ullán RV, Guerra SM, García-Estrada C, Vaca I, et al (2009) The transporter CefM involved in translocation of biosynthetic intermediates is essential for cephalosporin production. Biochemical Journal 418: 113 doi:10.1042/BJ20081180. 18840096 10.1042/BJ20081180

[11] Meijer WH, Gidijala L, Fekken S, Kiel JAKW, van den Berg MA, et al (2010) Peroxisomes are required for efficient penicillin biosynthesis in *Penicillium chrysogenum* . Appl Environ Microbiol 76: 5702–5709 doi:10.1128/AEM.02327-09. 20601503 10.1128/AEM.02327-09 PMC2935065

[12] Maggio-Hall LA, Wilson RA, Keller NP (2005) Fundamental contribution of β-oxidation to polyketide mycotoxin production *in planta* . Mol Plant Microbe Interact 18: 783–793 doi:10.1094/MPMI-18-0783. 16134890 10.1094/MPMI-18-0783

[13] Hong S-Y, Linz JE (2009) Functional expression and sub-cellular localization of the early aflatoxin pathway enzyme Nor-1 in *Aspergillus parasiticus* . Mycol Res 113: 591–601 doi:10.1016/j.mycres.2009.01.013. 19217941 10.1016/j.mycres.2009.01.013 PMC2765033

[14] Lee L-W, Chiou C-H, Klomparens KL, Cary JW, Linz JE (2004) Subcellular localization of aflatoxin biosynthetic enzymes Nor-1, Ver-1, and OmtA in time-dependent fractionated colonies of *Aspergillus parasiticus* . Arch Microbiol 181: 204–214 doi:10.1007/s00203-003-0643-3. 14722624 10.1007/s00203-003-0643-3

[15] Hong S-Y, Linz JE (2008) Functional expression and subcellular localization of the aflatoxin pathway enzyme Ver-1 fused to enhanced green fluorescent protein. Appl Environ Microbiol 74: 6385–6396 doi:10.1128/AEM.01185-08. 18757582 10.1128/AEM.01185-08 PMC2570292

[16] Chanda A, Roze LV, Linz JE (2010) A possible role for exocytosis in aflatoxin export in *Aspergillus parasiticus* . Eukaryot Cell 9: 1724–1727 doi:10.1128/EC.00118-10. 20870882 10.1128/EC.00118-10 PMC2976301

[17] Pestka JJ, Smolinski AT (2005) Deoxynivalenol: toxicology and potential effects on humans. Journal of Toxicology and Environmental Health, Part B 8: 39–69 doi:10.1080/10937400590889458. 10.1080/10937400590889458 15762554

[18] Alexander NJ, Proctor RH, McCormick SP (2009) Genes, gene clusters, and biosynthesis of trichothecenes and fumonisins in Fusarium. Toxin Reviews 28: 198–215 doi:10.1080/15569540903092142.

[19] McCormick SP, Harris LJ, Alexander NJ, Ouellet T, Saparno A, et al (2004) Tri1 in *Fusarium graminearum* encodes a P450 oxygenase. Appl Environ Microbiol 70: 2044–2051 doi:10.1128/AEM.70.4.2044-2051.2004. 15066795 10.1128/AEM.70.4.2044-2051.2004 PMC383062

[20] McCormick SP, Alexander NJ, Proctor RH (2006) Heterologous expression of two trichothecene P450 genes in *Fusarium verticillioides* . Can J Microbiol 52: 220–226 doi:10.1139/w05-124. 16604118 10.1139/w05-124

[21] McCormick SP, Alexander NJ, Proctor RH (2006) Fusarium Tri4 encodes a multifunctional oxygenase required for trichothecene biosynthesis. Can J Microbiol 52: 636–642 doi:10.1139/w06-011. 16917519 10.1139/w06-011

[22] Seong K-Y, Pasquali M, Zhou X, Song J, Hilburn K, et al (2009) Global gene regulation by Fusarium transcription factors Tri6 and Tri10 reveals adaptations for toxin biosynthesis. Molecular Microbiology 72: 354–367 doi:10.1111/j.1365-2958.2009.06649.x. 19320833 10.1111/j.1365-2958.2009.06649.x

[23] Menke J, Dong Y, Kistler HC (2012) *Fusarium graminearum* Tri12p influences virulence to wheat and trichothecene accumulation. Mol Plant Microbe Interact 25: 1408–1418 doi:10.1094/MPMI-04-12-0081-R. 22835271 10.1094/MPMI-04-12-0081-R

[24] Morton WM, Ayscough KR, McLaughlin PJ (2000) Latrunculin alters the actin-monomer subunit interface to prevent polymerization. Nature Cell Biology 2: 376–378 doi:10.1038/35014075. 10854330 10.1038/35014075

[25] Riedl J, Crevenna AH, Kessenbrock K, Yu JH, Neukirchen D, et al (2008) Lifeact: a versatile marker to visualize F-actin. Nature Methods 5: 605–607 doi:10.1038/nmeth.1220. 18536722 10.1038/nmeth.1220 PMC2814344

[26] Berepiki A, Lichius A, Read ND (2011) Actin organization and dynamics in filamentous fungi. Nature Reviews Microbiology 9: 876–887 doi:10.1038/nrmicro2666. 22048737 10.1038/nrmicro2666

[27] Seong K, Li L, Hou Z, Tracy M, Kistler HC, et al (2006) Cryptic promoter activity in the coding region of the HMG-CoA reductase gene in *Fusarium graminearum* . Fungal Genetics and Biology 43: 34–41 doi:10.1016/j.fgb.2005.10.002. 16377218 10.1016/j.fgb.2005.10.002

[28] Albermann S, Linnemannstöns P, Tudzynski B (2012) Strategies for strain improvement in *Fusarium fujikuro*i: overexpression and localization of key enzymes of the isoprenoid pathway and their impact on gibberellin biosynthesis. Appl Microbiol Biotechnol. doi:10.1007/s00253-012-4377-5. 10.1007/s00253-012-4377-5 22983595

[29] Coleman JJ, Mylonakis E (2009) Efflux in fungi: la pièce de résistance. PLoS Pathog 5: e1000486 doi:10.1371/journal.ppat.1000486. 19557154 10.1371/journal.ppat.1000486 PMC2695561

[30] Alexander NJ, McCormick SP, Hohn TM (1999) TRI12, a trichothecene efflux pump from *Fusarium sporotrichioides*: gene isolation and expression in yeast. Mol Gen Genet 261: 977–984. 10485289 10.1007/s004380051046

[31] McLaughlin JE, Bin-Umer MA, Tortora A, Mendez N, McCormick S, et al (2009) A genome-wide screen in *Saccharomyces cerevisiae* reveals a critical role for the mitochondria in the toxicity of a trichothecene mycotoxin. Proc Natl Acad Sci U S A 106: 21883–21888 doi:10.1073/pnas.0909777106. 20007368 10.1073/pnas.0909777106 PMC2799815

[32] Yang H-C, Pon LA (2002) Actin cable dynamics in budding yeast. Proc Natl Acad Sci U S A 99: 751–756 doi:10.1073/pnas.022462899. 11805329 10.1073/pnas.022462899 PMC117377

[33] Roze LV, Chanda A, Linz JE (2011) Compartmentalization and molecular traffic in secondary metabolism: A new understanding of established cellular processes. Fungal Genetics and Biology 48: 35–48 doi:10.1016/j.fgb.2010.05.006. 20519149 10.1016/j.fgb.2010.05.006 PMC2949687

[34] Gardiner DM, Kazan K, Manners JM (2009) Nutrient profiling reveals potent inducers of trichothecene biosynthesis in *Fusarium graminearum* . Fungal Genetics and Biology 46: 604–613 doi:10.1016/j.fgb.2009.04.004. 19406250 10.1016/j.fgb.2009.04.004

[35] Gaffoor I, Brown DW, Plattner R, Proctor RH, Qi W, et al (2005) Functional analysis of the polyketide synthase genes in the filamentous fungus *Gibberella zeae* (anamorph *Fusarium graminearum*). Eukaryot Cell 4: 1926–1933 doi:10.1128/EC.4.11.1926-1933.2005. 16278459 10.1128/EC.4.11.1926-1933.2005 PMC1287854

[36] Rosewich UL, Pettway RE, McDonald BA, Duncan RR, Frederiksen RA (1998) Genetic structure and temporal dynamics of a *Colletotrichum graminicola* population in a sorghum disease nursery. Phytopathology 88: 1087–1093 doi:10.1094/PHYTO.1998.88.10.1087. 18944821 10.1094/PHYTO.1998.88.10.1087

[37] Szewczyk E, Nayak T, Oakley CE, Edgerton H, Xiong Y, et al (2007) Fusion PCR and gene targeting in *Aspergillus nidulans* . Nature Protocols 1: 3111–3120 doi:10.1038/nprot.2006.405. 10.1038/nprot.2006.405 17406574

[38] Honda S, Selker EU (2009) Tools for fungal proteomics: multifunctional Neurospora vectors for gene replacement, protein expression and protein purification. Genetics 182: 11–23 doi:10.1534/genetics.108.098707. 19171944 10.1534/genetics.108.098707 PMC2674810

[39] Goswami RS, Kistler HC (2005) Pathogenicity and *in planta* mycotoxin accumulation among members of the *Fusarium graminearum* species complex on wheat and rice. Phytopathology 95: 1397–1404. 18943550 10.1094/PHYTO-95-1397

