# Supplementary figures and images for: Cellular Development Associated with Induced Mycotoxin Synthesis in the Filamentous Fungus Fusarium graminearum
Source: PLoS One. 2013 May 7;8(5):e63077. doi: 10.1371/journal.pone.0063077 (PMC3646755; doi:10.1371/journal.pone.0063077)

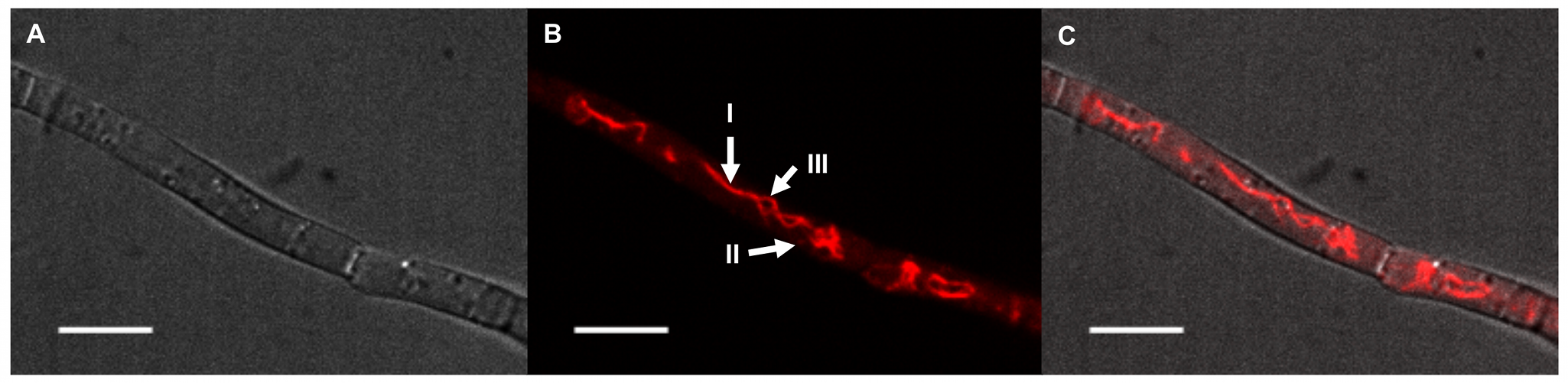

Supplement: Figure S1 — F-actin bound by Lifeact::RFP. Confocal bright field DIC (A), RFP (B), RFP and DIC overlay (C) images of Lifeact::RFP cells captured after 36 h of incubation in MM at 28°C in total darkness are shown. Actin cables (I); patches (II); and lariat-like structures (III) composed of actin cables are present. Scale bar = 10 µm. (TIF) [file pone.0063077.s001.tif]

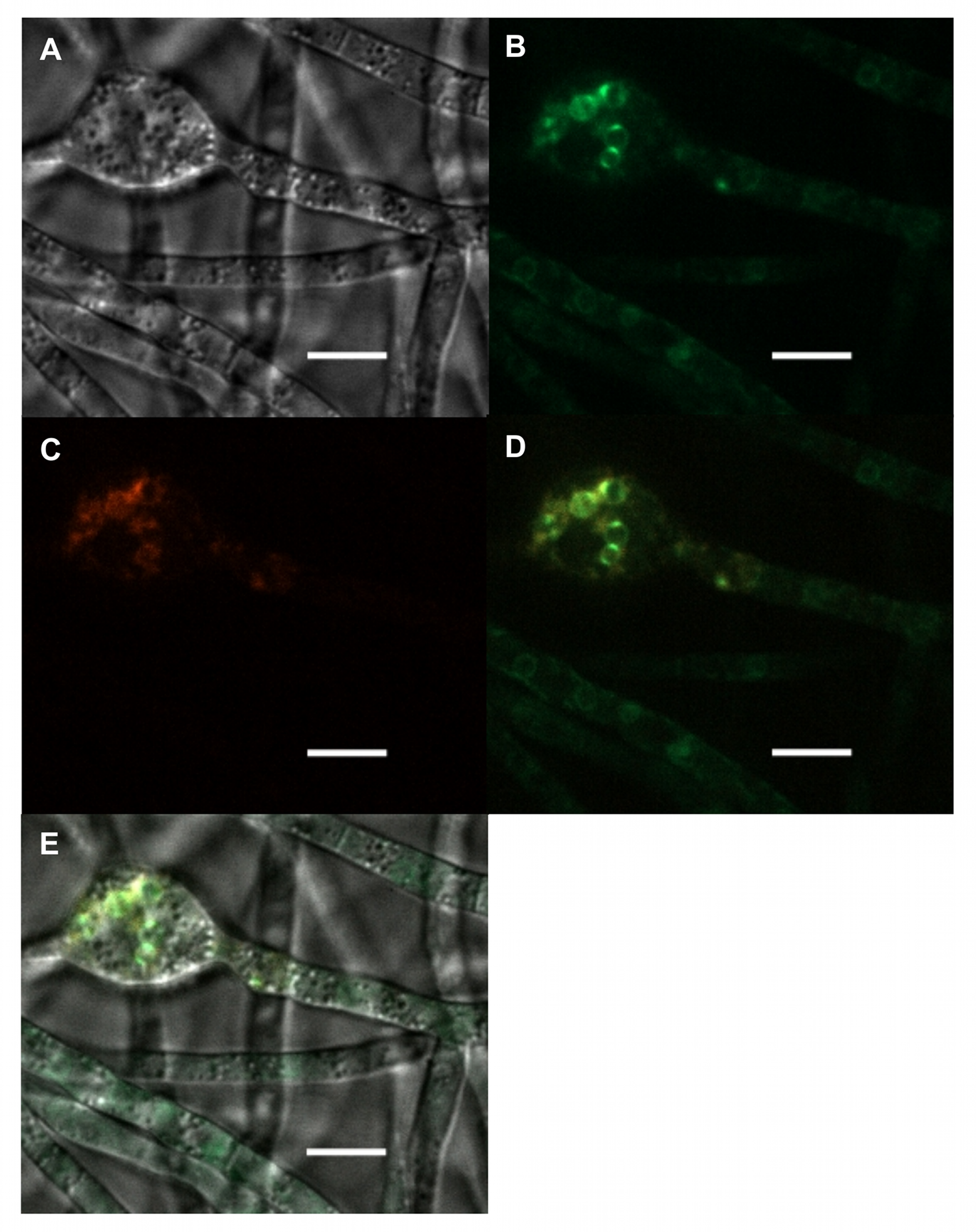

Supplement: Figure S2 — Visualization of Hmr1p and Tri4p under trichothecene biosynthesis inducing conditions. Expression of Hmr1p::GFP and Tri4p::RFP in strain PH-1Hmr1::GFP/Tri4::RFP under conditions where trichothecene biosynthesis occurs. Confocal bright field DIC (A); GFP (B); RFP (C); GFP and RFP overlay (D); and GFP, RFP and DIC overlay (E) images are shown of strain PH-1Hmr1::GFP/Tri4::RFP in TBI medium after 24 h incubation at 28°C in total darkness. Hmr1p::GFP expression is widespread among cells while Tri4p::RFP expression is limited. Scale bar = 10 µm. (TIF) [file pone.0063077.s002.tif]

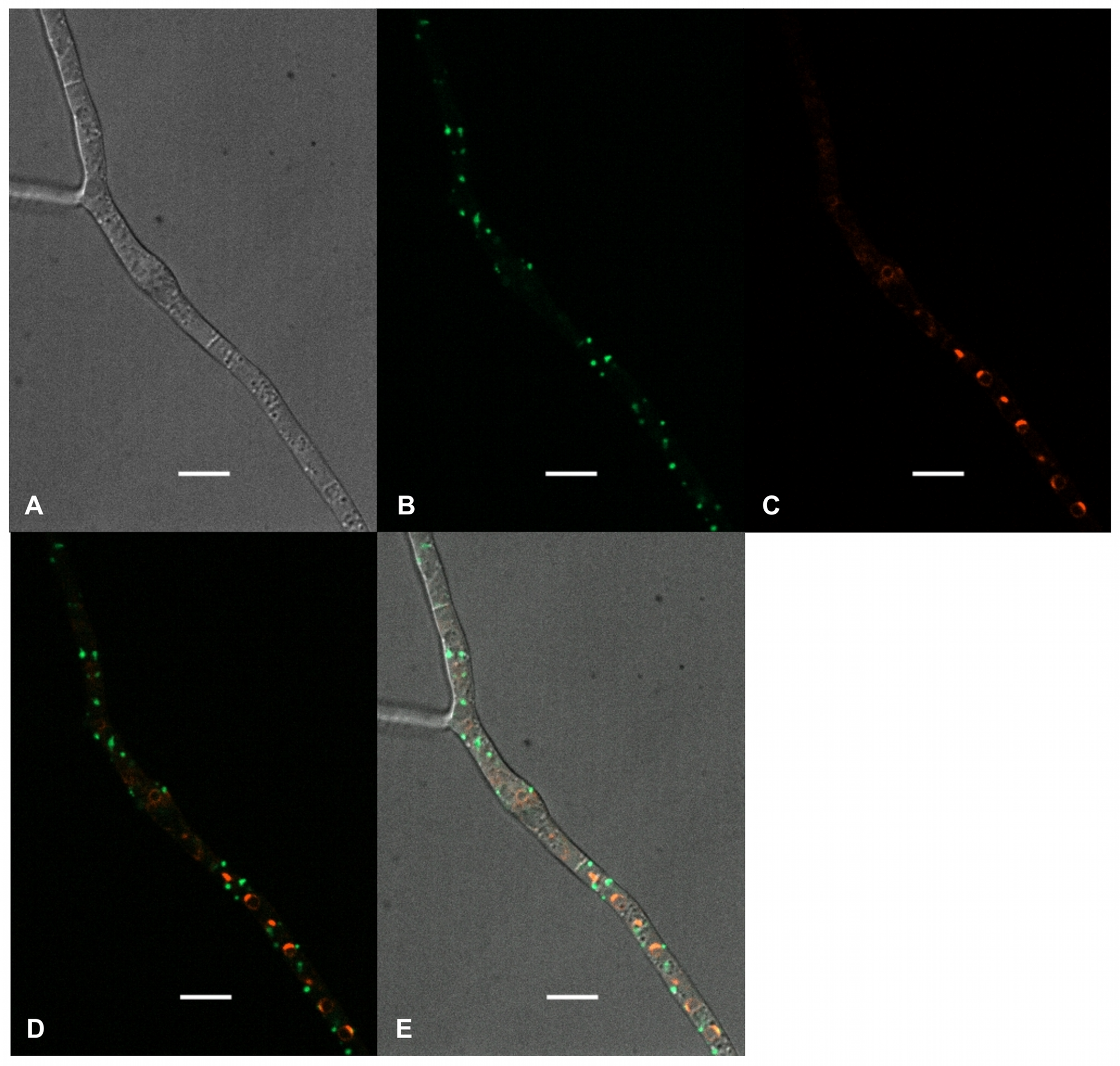

Supplement: Figure S3 — Visualization of Pex3p and Tri4p under trichothecene biosynthesis inducing conditions. Co-expression of Pex3p::GFP and Tri4p::RFP in strain PH-1Pex3::GFP/Tri4::RFP under conditions where trichothecene biosynthesis occurs. Confocal DIC (A); GFP (B); RFP (C); GFP and RFP overlay (D); and GFP, RFP and DIC overlay (E) images are shown of the strain in TBI medium after 24 h incubation at 28°C in total darkness. Pex3p::GFP and Tri4p::RFP localize exclusively to peroxisomes and toxisomes, respectively. Scale bar = 10 µm. (TIF) [file pone.0063077.s003.tif]
