# Supplementary material for: Cellular Development Associated with Induced Mycotoxin Synthesis in the Filamentous Fungus Fusarium graminearum
Source: PLoS One. 2013 May 7;8(5):e63077. doi: 10.1371/journal.pone.0063077 (PMC3646755; doi:10.1371/journal.pone.0063077)
Supplement: Table S1 — Oligonucleotides used for the synthesis of tagging constructs, Southern blots and PCR confirmation of GFP and RFP tagged strains. (DOCX) [file pone.0063077.s010.docx]

| Amplicons | Name | Sequence (5' to 3') |
| --- | --- | --- |
| 3' and 5' regions flanking the *Tri1* stop codon | Tri1GFPTagLF1F | GAAGAAGGGTGGAACACGAA |
|  | Tri1GFPTagLF2R | GCCTCCGCCTCCGCCTCCGCCGCCTCCGCCGTCATCCTGTACCAATTCCAATC |
|  | Tri1GFPTagRF3F | TCACCTAAATAGCTTGGCGTAATCATGGTCGTAGGAGGACGTCACAGTCTTGG |
|  | Tri1GFPTagRF4R | TCAGATTTGAGGCTCATAAGGTT |
| 3' and 5' regions flanking the *Tri12* stop codon | T12GFPTagLF1F | TTCCTACTTGTATTGTTGAGATGTTTCTA |
|  | T12GFPTagLF2R | GCTGTATCATCTGTTAGTGCTGTCTT |
|  | T12GFPTagRF3F | AATATCATAACTGTCCGGCATGCAGCTCG |
|  | T12GFPRF4R | AGGTAGGATGTTGGTAAGTTTATAGGTC |
| 3' and 5' regions flanking the *Hmr1* stop codon | Hmr1GFPTagLF1F | CATCATTCCTGGTGACGTTG |
|  | Hmr1GFPTagLF2R | GCCTCCGCCTCCGCCTCCGCCGCCTCCGCCTCGCTTTGACCTCTGGATG |
|  | Hmr1GFPTagRF3F | TCACCTAAATAGCTTGGCGTAATCATGGTCATGATGAGCGTTTGCTTTTG |
|  | Hmr1GFPTagRF4R | TATCGAAAAGGGCCAGGATA |
| 3' and 5' regions flanking the *Pex3* stop codon | Pex3GFPTagLF1F | TCCGACTGGCTTCACTTTCT |
|  | Pex3GFPTagLF2R | GCCTCCGCCTCCGCCTCCGCCGCCTCCGCCCGATTTGTTATCCATTGCTCGT |
|  | Pex3GFPTagRF3F | TCACCTAAATAGCTTGGCGTAATCATGGTCGGCGTATTTTGGTTGAGCAG |
|  | Pex3GFPTagRF4R | GCGAAGGTGTCAAGAACGAG |
| *GFPhyp* constructs used to tag *Tri1, Hmr1 and Pex3* | GFPTagFwd | GGCGGAGGCGGCGGAGGCGGAGGCGGAGGC |
|  | GFPTagRev | GACCATGATTACGCCAAGCTATTTAGGTGA |
| *GFPhyp* constructs used to tag*Tri12* | GFPFwLFTri12Seq | AAGACAGCACTAACAGATGATACAGCGGCGGAGGCGGCGGAGGCGGAGGCGGAGGC |
|  | GFPRwRFTri12Seq | CGAGCTGCATGCCGGACAGTTATGATATTGACCATGATTACGCCAAGCTATTTAGGTGA |
| 3' and 5' regions flanking the *Tri4* stop codon | TRI4RFPTagLF1F | CGTGTGGCTACTCAGGAGAAC |
|  | TRI4RFPTagLF2R | CTCTTCGCCCTTAGACACCATCGAGCCTCCGCCTCCGCCTCCGCCGCCTCCGCCCAAAGCCTTGAGAACCTTGA |
|  | TRI4RFPTagRF3F | AGCATGCCCTGCCCCTGATCTAGAGCACATGGCGCATCTGACAAACTGTC |
|  | TRI4RFPTagRF4R | TGGTGAAGACACATAGTCAACAGA |
| *RFPnat1* construct used to tag *Tri4* | RFPTagFwd | GGCGGAGGCGGCGGAGGCGGAGGCGGAGGCTCGATGGTGTCTAAGGGCGAAGAG |
|  | RFPTagRev | ATGTGCTCTAGATCAGGGGCAGGGCATGCT |
| *Lifeact* construct synthesis | LifeactF | CCGTGACCACTGAACTACACTAGTCT |
|  | LifeactR | ATGTGCTCTAGATCAGGGGCA |

| Amplicons | Name | Sequence (5' to 3') |
| --- | --- | --- |
| Probes for Southern blots | Tri1South5P | GCTCGCGAACTAATCACTCC |
|  | Tri1South3P | AATTCCAATCGCAGACAAGG |
|  | Tri4South5P | GAAGCGCCTCCCTTACTCTT |
|  | Tri4South3P | CGGTGTGCATGAAATAGGTG |
|  | Tri12South5P | TTCCCAACATTCTCCAGGAC |
|  | Tri12South3P | CCTATCCAGTCGAGCTTTGC |
|  | GFPSouth5P | CACATGAAGCAGCACGACTT |
|  | GFPSouth3P | GCACCAAGCAGCAGATGATA |
|  | RFPSouth5P | CTTCATGTACGGCAGCAGAA |
|  | RFPSouth3P | GCTCGTCCATGCCATTAAGT |
| PCR amplicons used for confirmation of *Hmr1* and *Pex3* tagging | Hmr1GFPTagTestExtF | TTAGCGTCATGTCCAACGAG |
|  | Pex3GFPTestExtF | GAAAGTCCGACTGGCTTCAC |
|  | GFPTestR | GAACTTCAGGGTCAGCTTGC |
|  | hphGFPTestR | GATGTTGGCGACCTCGTATT |
